# Trends in Social Norms Toward Cigarette Smoking and E-cigarette Use Among U.S. Youth Between 2015 and 2021

**DOI:** 10.1093/ntr/ntaf120

**Published:** 2025-06-30

**Authors:** Giang T Vu, Tianze Sun, Wayne Hall, Jason P Connor, Phong Thai, Coral Gartner, Janni Leung, Gary Chan

**Affiliations:** National Centre for Youth Substance Use Research (NSYCUR), School of Psychology, The University of Queensland, Brisbane, Australia; National Centre for Youth Substance Use Research (NSYCUR), School of Psychology, The University of Queensland, Brisbane, Australia; National Centre for Youth Substance Use Research (NSYCUR), School of Psychology, The University of Queensland, Brisbane, Australia; National Centre for Youth Substance Use Research (NSYCUR), School of Psychology, The University of Queensland, Brisbane, Australia; Discipline of Psychiatry, The University of Queensland, Brisbane, Australia; Queensland Alliance for Environmental Health Sciences (QAEHS), The University of Queensland, Brisbane, Australia; NHMRC Centre of Research Excellence on Achieving the Tobacco Endgame, School of Public Health, The University of Queensland, Brisbane, Australia; National Centre for Youth Substance Use Research (NSYCUR), School of Psychology, The University of Queensland, Brisbane, Australia; National Centre for Youth Substance Use Research (NSYCUR), School of Psychology, The University of Queensland, Brisbane, Australia

## Abstract

**Introduction:**

This study examines trends in social norms toward cigarette smoking and e-cigarette use among US youth during 2015-2021, focusing on descriptive interpersonal norms (friends’ behavior) and injunctive norms at interpersonal (perceived important others’ negative view) and societal level (perceived public disapproval).

**Methods:**

Respondents were youth aged 12 to 17 from the Population Assessment of Tobacco and Health Study of the United States, Wave 3 (2015-2016) to Wave 6 (2021). Logistic regression models that adjusted for demographics and participation effects assessed norm changes over time and their association with use status in Wave 6.

**Results:**

Between 2015 and 2021, the probability of having friends who smoked cigarettes decreased (26.1% vs. 7.9%, adjusted odds ratio [aOR] = 0.81 [95% confidence interval = 0.72 to 0.91]), while having friends who use e-cigarettes generally decreased (31.6% vs. 22.3%, aOR = 0.46, [0.37-0.58]) despite an increase in 2018-2019. Perceived negative views from important others remained stable for both products during 2015-2019, peaked in 2020 (85.2% and 86.2%) before declining slightly in 2021. Perceived public disapproval increased to a peak in 2020 for both products (73.3% to 84.2% for cigarettes and 55.4% to 77.5% for e-cigarettes). In 2021, having friends who used e-cigarettes was associated with current e-cigarette use (relative risk ratio [RRR] = 15.07 [9.94-22.85]) and current dual use (RRR = 3.38 [1.41-8.13]), while important others’ negative view toward e-cigarette use reduced the likelihood of current e-cigarette use (RRR = 0.3 [0.2-0.44]).

**Conclusions:**

Among US youth during 2015-2021, norms consistently indicated denormalization of cigarette smoking. e-cigarette norms showed greater variability, particularly during the coronavirus disease 2019 (COVID-19) pandemic.

**Implications**

The persistent gap between interpersonal and societal disapproval, especially for e-cigarettes, highlights the need for strengthening societal-level interventions. The strong protective effect of important others’ negative views and the powerful influence of peer behavior suggest that prevention strategies should simultaneously address multiple social contexts. Programs engaging parents and educators should be complemented by campaigns to correct misperceptions about e-cigarette prevalence and enhance societal disapproval, particularly as youth social contexts continue to evolve post-pandemic.

## Introduction

According to the Theory of planned behavior, social norms play a major role in shaping human behavior.^1^ Theory of planned behavior posits that an individual’s actions are influenced by both descriptive and injunctive social norms.^2,3^ Descriptive norms refer to the perception of others’ observed or inferred behavior (eg friends smoking or vaping). Injunctive norms reflect the perception of whether others approve or disapprove of the behavior (eg important others disapprove of smoking).^3,4^

The social ecological model of health behavior suggests that one’s behavior would be influenced by factors at multiple levels from intrapersonal, to interpersonal involving direct social connections such as friends and family, to broader societal level covering community and policy environments.^5^ Thus, both descriptive and injunctive norms operate at both proximal social level (friends, family) and distal societal level (broader community).^6^ Studies have demonstrated how these norms influenced tobacco use: smoking initiation and cessation were more common among those whose close social networks included people engaging in similar behaviors (descriptive interpersonal norms),^7,8^ while norms against smoking from significant others and the society (injunctive interpersonal and societal norms) were significantly associated with intention to quit smoking.^9^ Similar patterns of influence emerge for e-cigarette use, where initiation was influenced by both perceived use among friends or in public,^6,10^ and perceived societal approval.^6^

Tobacco control policies, such as smoke-free laws and public health campaigns, have contributed to the denormalization of smoking by shifting perceptions of smoking as common and acceptable to less common and disapproved.^11^ This shift has played a critical role in reducing smoking prevalence and promoting cessation.^12–14^ However, the increasing popularity of e-cigarettes among youth raises concerns about the potential renormalization of tobacco smoking,^15,16^ which could lead to the reversal of the long-term trend of decreasing cigarette smoking among youth.^6^

Psychology research proposes that youth may be more susceptible to social influence than adults.^17–19^ Both quantitative and qualitative research on youth cigarette smoking and e-cigarette use has demonstrated the association between norms and use among this population. Youth who report having friends or family smoking cigarettes^20,21^ or using e-cigarettes^20,22–24^ were more likely to smoke or vape. Perceived positive attitude toward use from close social network^20,24^ or the society^21^ was associated with a higher likelihood of use, while disapproval from friends and family deterred use.^25–27^ Some studies documented the association between cross-product norms and use: having friends or family who smoke and higher odds of trying e-cigarettes,^28^ having friends using e-cigarettes and higher odds of smoking initiation,^29^ or greater intention to quit smoking.^10^

These studies of the importance of social norms in youth cigarette smoking and e-cigarette use primarily examined norms at single timepoints. Understanding how these norms evolve over time could provide valuable insights for public health interventions, especially those addressing the renormalization concern.

Using data from the Population Assessment of Tobacco and Health (PATH) study, a nationally representative longitudinal cohort study of U.S. youth and adults, this paper aims to:

1. Estimate the trends of social norms toward cigarette smoking and e-cigarette use among youth in the United States.
2. Quantify the association between social norms and cigarette smoking and e-cigarette use in participants at the most current wave of data.

## Methods

### Design

This study employed a series of cross-sectional analyses using data from the PATH Study, a representative, longitudinal cohort study of civilian, non-institutionalized youth and adults in the United States. Cross-sectional analyses were chosen to examine age group changes in attitudes over time, rather than individual changes as participants aged.

### Participants and Sample

Data for this analysis were sourced from the PATH youth cohort (aged 12 to 17) Wave 3 (W3) to W6.

Data collection for W3 occurred between October 19, 2015 and October 23, 2016 (*N* = 11 814); for W4, between December 1, 2016 and January 3, 2018 (*N* = 14 793); for W4.5, between December 1, 2017 and December 1, 2018 (*N* = 12 918); for W5, between December 1, 2018 and November 30, 2019 (*N* = 11 976); for W5.5, between July 3, 2020 and December 31, 2020 (*N* = 7129); and for W6, between March 1 and November 30, 2021 (*N* = 5585).

The PATH Study’s weighted response rates for the youth cohort at W1 were 83.3%, 79.5%, 74.6%, 72.3%, and 56.6% at W3, W4, W4.5, W5, and W6, respectively. The PATH Study’s weighted response rates for the W4 cohort (replenishment cohort, designed to supplement the W1 sample) were 78.7%, 89.1%, 83.5%, 66.8%, and 63.6% at W4, W4.5, W5, W5.5 and W6, respectively. Further information about the PATH study is available elsewhere.^30^

### Measures

#### Outcome Variables

##### Social Norms Toward Cigarette Smoking and e-Cigarette Use.

One item was used to assess descriptive norms at the proximal interpersonal level for each product type. Participants were asked to report the number of their best friends who used a specific product, that is, “How many of your best friends smoke cigarettes/ use e-cigarettes or other electronic nicotine products?.” Responses were recoded as None, At least a few (A few/ Some/ Most/ All).

Two items were used to assess injunctive norms at both the proximal interpersonal level and distal societal level for each product type. Participants were asked to describe the views of people important to them toward the use of a specific product (injunctive interpersonal norms), that is, “Thinking about the people who are important to you, how would you describe their views on the following: Cigarette smoking/ Using e-cigarettes or other electronic nicotine products?.” Responses were recoded as Negative (Negative, Very negative), Others (Very positive, Positive, Neither positive nor negative). For each product type, participants were also asked whether they thought most people disapprove of the use of the specific product (injunctive societal norms), for example, “In general, do you think most people disapprove of cigarettes smoking/ using e-cigarettes or other electronic nicotine products?.” Responses were coded as Disapprove (Definitely yes, Probably yes), Others (Probably not, Definitely not).

#### Predictor Variables

Survey wave: 3 (2015-2016), 4 (2016-2017), 4.5 (2017-2018), 5 (2018-2019), W5.5 (2020), 6 (2021).

#### Covariates

Demographic variables included self-reported age group, designated sex at birth, ethnicity, and annual household income.

Time in the sample was the number of waves the participant was involved in (1-6), included to control for potential participation effects.

#### Cigarette Smoking and e-Cigarette Use

For each product type, participants were asked the following three questions about their use of that respective product in the past 30 days, past 12 months, and lifetime: “In the past 30 days, on how many days did you smoke cigarettes/ use an electronic nicotine product?,” “In the past 12 months, have you smoked a cigarette, even one or two puffs/ used an electronic nicotine product, even one or two times?,” and “Have you ever tried cigarette smoking, even one or two puffs/ used an electronic nicotine product, even one or two times?.” Participants were then classified into five mutually exclusive groups representing their e-cigarette and cigarette use: (1) current (past 30 days) e-cigarette use only, (2) current cigarette smoking only, (3) current e-cigarette use and cigarette smoking (dual current use), (4) never use either product, and (5) non-current use of either or both products.

### Statistical Analyses

Weights were applied to align the sample with the US population, and the *svy* command was used to adjust for the complex survey design. Sample characteristics of participants were estimated on weighted data.

To address Aim 1, binary logistic regression models using Generalized Estimating Equations were adopted to regress each outcome measure on time (waves), with demographic variables and time in the sample included as covariates to examine the change in norms over time. Predicted probabilities of each outcome at each wave were estimated from these models using Stata’s margins command. This was done to account for the influence of time in the sample on the outcome when assessing trend.

To address Aim 2, bivariate and multivariate multinomial logistic regression models were used to regress use types on the set of outcome variables, adjusting for demographic variables and time in the sample, for participants using the latest wave (Wave 6). For these regressions, current cigarette smoking was excluded due to the small number of observations (*n* = 17, 0.30%).

For all outcome variables, the “Do not know” and “Refused” responses were coded as missing. The percentage of missing data ranged from 0.35% to 5.85% across variables. Multiple imputations using iterated chained equations algorithm were used to fill in missing values and 20 datasets were imputed, assuming data were missing at random.^31^

All analyses were conducted using Stata v16. Statistical significance was reported for *p* < .05.

## Results

### Sample Characteristics

A total of 64 215 observations was included in this study (W3: *n* = 11 814; W4: *n* = 14 793; W4.5: *n* = 12 918; W5: *n* = 11 976; W5.5: *n* = 7129; W6: *n* = 5585). Sex, race, and annual household income distribution were similar across waves with females accounting for 48.6% on average, White ethnicity (67.9% on average) and annual household income of $100 000 or more (33.2% on average) were most highly reported. The 15–17-year-old group comprised the majority of the participants in W5.5 and W6 (Table 1).

**Table 1.** Descriptive Statistics of Participants

	Wave 3	Wave 4	Wave 4.5	Wave 5	Wave 5.5	Wave 6
* n*	*11 814*	*14 793*	*12 918*	*11 976*	*7129*	*5585*
Sex						
* Male*	51.43%	51.43%	51.52%	51.38%	51.13%	51.26%
* Female*	48.57%	48.57%	48.48%	48.62%	48.87%	48.74%
Age (years old)						
* 12-14*	50.42%	48.33%	48.02%	47.52%	39.11%	28.33%
* 15-17*	49.58%	51.67%	51.98%	52.48%	60.89%	71.67%
Race						
* White*	69.07%	68.47%	68.41%	67.43%	67.34%	66.71%
* Black*	15.52%	15.99%	15.47%	15.72%	15.27%	15.33%
* Others*	15.42%	15.54%	16.12%	16.85%	17.39%	17.95%
Annual Household Income						
* Less than $10 000*	7.29%	7.63%	6.23%	6.52%	5.16%	5.32%
* $10 000 to $24,99*	15.47%	14.48%	13.68%	13.33%	12.09%	10.45%
* $25 000 to $49,99*	22.23%	22.04%	21.12%	21.04%	19.88%	19.22%
* $50 000 to $99,99*	26.67%	26.77%	26.71%	25.75%	25.81%	25.88%
* $100 000 or more*	28.34%	29.08%	32.26%	33.37%	37.06%	39.13%
Current cigarette smoking	3.21%	3.22%	2.68%	2.32%	1.29%	1.00%
Current e-cigarette use	4.09%	4.41%	6.96%	8.82%	4.47%	5.66%
Time in sample						
* 1 wave*	100.00%	32.78%	15.75%	17.38%	1.30%	1.02%
* 2 waves*	0.00%	67.22%	28.82%	14.75%	21.94%	0.86%
* 3 waves*	0.00%	0.00%	55.43%	25.64%	19.94%	25.85%
* 4 waves*	0.00%	0.00%	0.00%	42.22%	28.05%	24.26%
* 5 waves*	0.00%	0.00%	0.00%	0.00%	28.78%	29.08%
* 6 waves*	0.00%	0.00%	0.00%	0.00%	0.00%	18.94%

Data are weighted.

The weighted prevalence of current cigarette smoking decreased steadily between W3 (2015-2016) and W6 (2021). Current e-cigarette use increased gradually from 4.1% at W3 (2015-2016) to peak at 8.8% at W5 (2019-2020) before falling to 4.5% at W5.5 (2019) and recovering to 5.7% at W6 (2021) (Table 1).

The weighted average time in the sample of participants was 2.4 waves.

### Descriptive Interpersonal Norms

#### Trend

**Figure 1.**
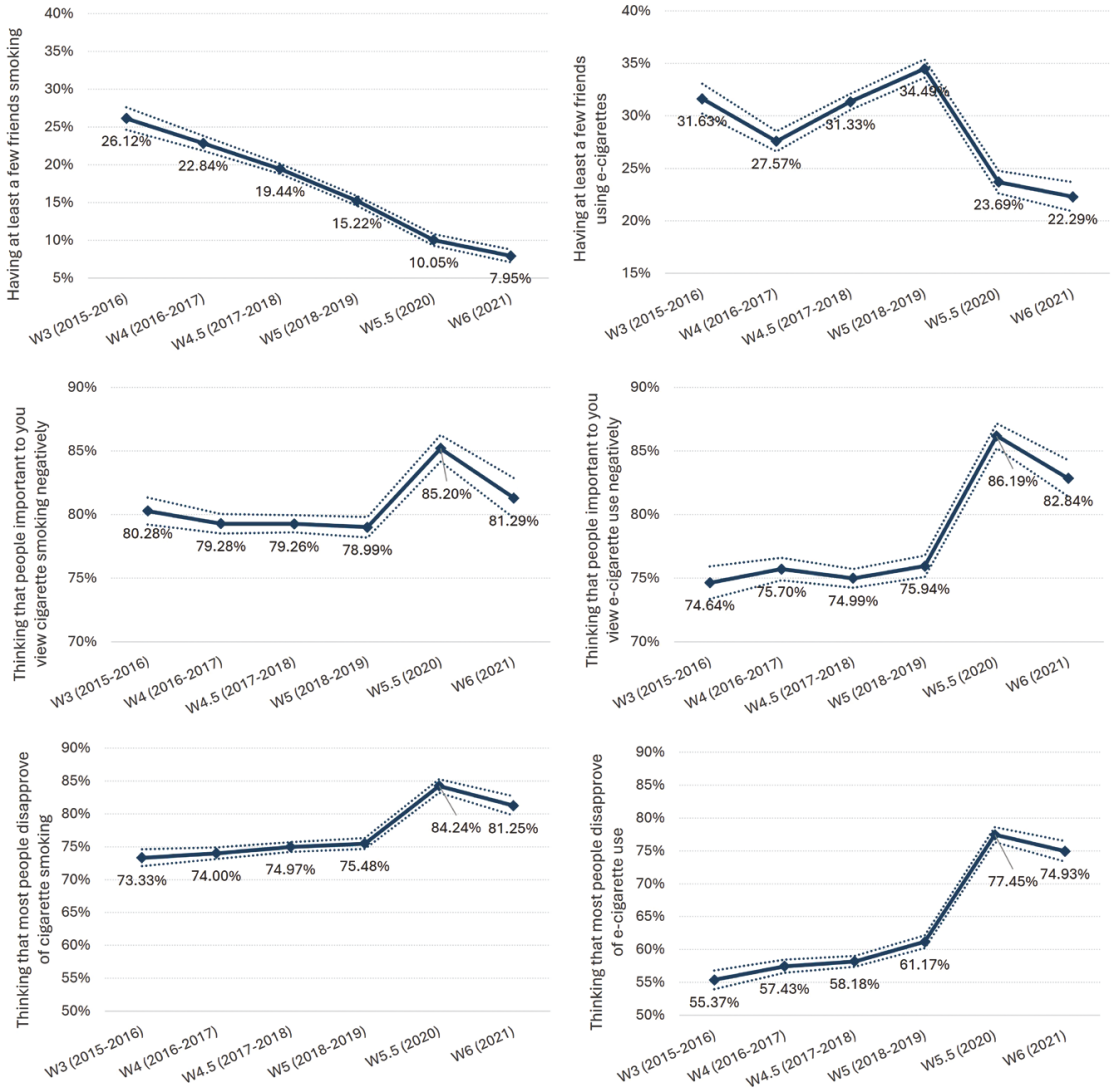
Predicted probabilities and 95% confidence interval of having at least a few friends smoking, having at least a few friends using e-cigarettes (descriptive interpersonal norms), thinking that people important to you view cigarette smoking negatively, thinking that people important to you view e-cigarette use negatively (injunctive interpersonal norms), thinking that most people disapprove of cigarette smoking, thinking that most people disapprove of e-cigarette use (injunctive societal norms), by wave (W). Data are from binary logistic regression analyses, adjusted for age, sex, race, income and time in sample, and weighted (*N* = 64 215 observations from 20 850 unique participants). Dotted lines denoting 95% confidence interval.

The probability of youth who had at least a few friends who smoked steadily declined from 26.1% to 7.9%, with significantly lower odds in recent waves compared to 2015-2016 (2020: adjusted odds ratio [aOR] = 0.80 [95% confidence interval [CI] = 0.72 to 0.90]; 2021: aOR = 0.81 [0.72-0.91], both *p* < .001) (Figure 1, Table 2). The probability of having at least a few friends who used e-cigarettes showed more variation, initially decreasing from 31.6% to 27.6% (2016-2017), then increasing to 34.5% (2018-2019), before declining to 23.7% in 2020 and 22.3% in 2021 (2020: aOR = 0.53 [0.44-0.63]; 2021: aOR = 0.46 [0.37-0.58], both *p* < .001).

**Table 2.** Associations Between Social Norms and Time (Adjusted for Covariates)

	Descriptive interpersonal norms				Injunctive interpersonal norms				Injunctive societal norms			
	Having at least a few friends smoking		Having at least a few friends using e-cigarettes		Thinking that people important to you view cigarette smoking negatively		Thinking that people important to you view e-cigarette use negatively		Thinking that most people disapprove of cigarette smoking		Thinking that most people disapprove of e-cigarette use	
Time	AOR (95% CI)	*p*-value	AOR (95% CI)	*p*-value	AOR (95% CI)	*p*-value	AOR (95% CI)	*p*-value	AOR (95% CI)	*p*-value	AOR (95% CI)	*p*-value
Wave 3 (2015-2016)	1.00		1.00		1.00		1.00		1.00		1.00	
Wave 4 (2016-2017)	**0.77 (0.7, 0.84)**	**<.001**	**0.73 (0.67, 0.79)**	**<.001**	**0.92 (0.84, 1)**	**.04**	1.09 (1.01, 1.18)	.05	1.05 (0.98, 1.13)	.21	**1.12 (1.05, 1.2)**	**.01**
Wave 4.5 (2017-2018)	**0.56 (0.5, 0.64)**	**<.001**	0.98 (0.88, 1.1)	.69	0.92 (0.82, 1.02)	.1	1.03 (0.93, 1.14)	.62	**1.13 (1.02, 1.24)**	**.02**	**1.17 (1.07, 1.27)**	**<.001**
Wave 5 (2018-2019)	**0.37 (0.31, 0.43)**	**<.001**	**1.25 (1.08, 1.43)**	**.01**	0.89 (0.78, 1.03)	.1	1.11 (0.97, 1.26)	.14	**1.17 (1.04, 1.32)**	**.02**	**1.37 (1.24, 1.52)**	**<.001**
Wave 5.5 (2019-2020)	**0.19 (0.15, 0.23)**	**<.001**	**0.53 (0.44, 0.63)**	**<.001**	**1.66 (1.38, 2)**	**<.001**	**2.83 (2.38, 3.37)**	**<.001**	**2.44 (2.08, 2.86)**	**<.001**	**3.74 (3.27, 4.28)**	**<.001**
Wave 6 (2021)	**0.14 (0.11, 0.17)**	**<.001**	**0.46 (0.37, 0.58)**	**<.001**	1.11 (0.89, 1.38)	.4	**2 (1.63, 2.47)**	**<.001**	**1.85 (1.53, 2.24)**	**<.001**	**3.14 (2.67, 3.69)**	**<.001**

Data are from binary logistic regression analyses, adjusted for age, sex, race, income and time in sample and weighted.

Bold values indicate statistical significance at *p* < .05.

Abbreviations: AOR = adjusted odds ratio, 95% CI = 95% confidence interval.

Unadjusted analyses showed some different patterns for descriptive interpersonal norms. Having at least a few friends who smoke showed significant decreases across all waves in unadjusted models (Odds Ratio [OR] range: 0.77-0.14, all *p* < .001), while for having at least a few friends who use e-cigarettes, unadjusted analyses suggested increasing odds over time (OR range: 1.15-7.45, all *p* < .01) (Table S1).

#### Association With Cigarette Smoking and e-Cigarette Use in Participants at W6 (2021)

When never use of either product was the reference group, having at least a few friends smoking was significantly positively associated with current dual use, while having at least a few friends who used e-cigarettes was significantly positively associated with any use (current e-cigarette use: relative risk ratio [RRR] = 15.07 [95% CI = 9.94 to 22.85], *p* < .001; current dual use: RRR = 3.38 [1.41-8.13, *P* = .01]; non-current use: RRR = 3.02 [2.47-3.69, *p* < .001]) (Table 3).

**Table 3.** Association Between Social Norms and e-Cigarette and Cigarette Use in Participants at W6

	Current e-cigarette use		Current dual use		Non-current use of either or both product	
	RRR (95% CI)	*p*-value	RRR (95% CI)	*p*-value	RRR (95% CI)	*p*-value
**Descriptive interpersonal norms**						
Having at least a few friends smoking (ref. None)	1.39 (0.99, 1.94)	.06	**13.96 (5.96, 32.71)**	**<.001**	1.09 (0.84, 1.43)	.54
Having at least a few friends using e-cigarettes (ref. None)	**15.07 (9.94, 22.85)**	**<.001**	**3.38 (1.41, 8.13)**	**.01**	**3.02 (2.47, 3.69)**	**<.001**
**Injunctive interpersonal norms**						
Thinking that people important to you view cigarette smoking negatively (ref. Others)	1.31 (0.83, 2.07)	.26	0.5 (0.17, 1.44)	.2	0.86 (0.61, 1.21)	.38
Thinking that people important to you view e-cigarette use negatively (ref. Others)	**0.3 (0.2, 0.44)**	**<.001**	**0.36 (0.14, 0.95)**	**.04**	**0.63 (0.46, 0.86)**	**.01**
**Injunctive societal norms**						
Thinking that most people disapprove of cigarette smoking (ref. Others)	0.93 (0.6, 1.45)	.75	1.22 (0.49, 3.09)	.68	1.03 (0.78, 1.36)	0.88
Thinking that most people disapprove of e-cigarette use (ref. Others)	1.44 (1.01, 2.07)	.05	1.06 (0.5, 2.26)	.89	1.21 (0.95, 1.54)	.15

Only Wave 6 participants (*n* = 5585) were included in this multivariate logistic regression analysis, where use types (current e-cigarette use: *n* = 280, current dual use: *n* = 45, non-current use: *n* = 735, never use of either product: *n* = 4493) were regressed on the set of descriptive and injunctive norms, adjusted for age, sex, race, income and time in sample. Current cigarette smoking (*n* = 17) was excluded due to small number of observations. Never use of either product was used as reference group.

Bold values indicate statistical significance at *p* < .05.

Abbreviations: 95% CI = 95% confidence interval; RRR = relative risk ratio.

### Injunctive Interpersonal Norms

#### Trend

The probability of youth thinking that people important to them viewed cigarette smoking negatively fluctuated around 79%-80% during 2015-2019, then peaked at 85.2% in 2020 (aOR = 1.66 [1.38-2.00], *p* < .001), before decreasing to 81.3% in 2021 (Figure 1, Table 2). Similarly, the probability of thinking that people important to them viewed e-cigarette use negatively remained around 75% until 2020, when it increased to 86.2% (aOR = 2.83, [2.38-3.37], *p* < .001), then decreased to 82.8% in 2021.

The unadjusted model showed a similar trend (Table S1).

#### Association With Cigarette Smoking and e-Cigarette Use in Participants at W6 (2021)

No significant association was found between use and injunctive interpersonal norms toward smoking. Thinking that people important to you view e-cigarette use negatively was significantly negatively associated with any use (compared to never use, current e-cigarette use: RRR = 0.3, [0.2-0.44], *p* < .001; current dual use: RRR = 0.36 [0.14-0.95], *P* = .04; non-current use: RRR = 0.63 [0.46-0.83], *P* = .01) (Table 3).

### Injunctive Societal Norms

#### Trend

The probability of youth thinking that most people disapproved of cigarette smoking increased from 73.3% to 84.2% in 2020 (aOR = 2.44 [2.08-2.86], *p* < .001), then decreased to 81.2% in 2021 (Figure 1, Table 2). The probability of thinking that most people disapproved of e-cigarette use showed a more pronounced increase, rising from 55.4% to 77.5% in 2020 (aOR = 3.74 [3.27-4.28], *p* < .001), before slightly decreasing to 74.9% in 2021 (Figure 1, Table 2).

The patterns were generally similar with the unadjusted model, particularly for the significant increases in 2020 (Table S1).

#### Association With Cigarette Smoking and e-Cigarette Use in Participants at W6 (2021)

No significant association was found between cigarette or e-cigarette use and injunctive societal norms.

## Discussion

This study examined trends in different types of social norms about cigarette smoking and e-cigarette use among US youth between 2015 and 2021. The findings reveal distinct patterns across different norm types and products that have important implications for tobacco control policy and practice.

Descriptive interpersonal norms for cigarette smoking suggested consistent denormalization of smoking, with the proportion of youth who reported having friends who smoke declining steadily from 26.1% to 7.9% over the 2015-2021 period. This trend aligns with broader tobacco control successes in reducing youth smoking rates^32,33^ and suggests that youth social environments have moved away from smoking thanks to decades of comprehensive tobacco control measures, including price increases, smoke-free laws, banning sales to minors and youth-targeted smoking prevention strategies like school-based education programs and mass media campaigns.^34–37^ The sustained decrease in friends who smoke, even after controlling for time-in-sample effects, indicates genuine denormalization rather than just reporting bias.

In contrast, descriptive interpersonal norms for e-cigarettes showed more complex patterns. The fluctuation in having friends who vape—peaking at 34.5% in 2018-2019 before declining to 22.3% in 2021—mirrors documented trends in youth e-cigarette use.^38^ This pattern likely reflects the interplay of several factors: the introduction of youth-appealing e-cigarette devices like the “pod mods” (notably JUUL); the fact that disposables offered a wide range of flavors in stylish designs which could be purchased at relatively low prices and used discreetly; and aggressive marketing targeting youth during 2015-2019.^39–41^ Regulatory responses such as flavor restrictions,^42^ and the e-cigarette or vaping associated lung injury or EVALI outbreak in late 2019 may also have played a role.^43,44^ The sharp decline in 2020 may reflect regulatory impacts as well as the  disruptions to social networks and reduced product access related to the coronavirus disease 2019 (COVID-19).^45,46^

Injunctive norms showed different patterns from descriptive norms. The notable increase in perceived disapproval of both products in 2020 (reaching 85.2% for smoking and 86.2% for vaping) suggests that public health messaging and policy actions may have strengthened anti-tobacco sentiment during this period. This increase may also partly be a result of the heightened concerns about the potential association between cigarette smoking and e-cigarette use and the increased risk of COVID-19 in youth.^47^ The pandemic also disrupted typical youth social interactions through school closures, social distancing, and increased time spent at home with family, potentially amplifying the influence of parental views while reducing peer influences. However, the subsequent decline in perceived disapproval in 2021 (to 81.3% for smoking and 82.8% for vaping) raises questions about the sustainability of these shifts. This decline may reflect the resumption of in-person social activities, reduced parental supervision as pandemic restrictions eased, and a possible “rebound effect” in youth social behaviors.^48^ The return to pre-pandemic social contexts might have diluted the heightened health consciousness and family-centered influences of 2020, highlighting the role of the social environment in shaping tobacco-related norms. In addition, the consistently higher disapproval from important others compared to perceived societal disapproval, particularly for e-cigarettes suggests that while immediate social circles, for example, parents and teachers, may maintain strong anti-tobacco stances, broader social messaging is mixed, potentially reflecting industry marketing and social media influences.^49,50^

These findings have several policy implications. First, the success in denormalizing smoking suggests the need to maintain and strengthen existing tobacco control measures. Second, the more volatile e-cigarette norms indicate a need for consistent regulatory approaches, particularly around youth-oriented marketing and flavors. Third, the gap between interpersonal and societal disapproval highlights opportunities for multi-level interventions that align important others and broader societal disapproval of cigarette smoking and e-cigarette use.

Community-level programs should engage parents, teachers, and youth leaders in consistent messaging about cigarette smoking and e-cigarette use, build capacity for peer-led prevention initiatives, create youth-driven counter-marketing campaigns, and foster partnerships between schools, public health organizations, and youth-serving organizations. At the broader societal level, campaigns should focus on correcting misperceptions about the prevalence of smoking and e-cigarette use among youth. These should feature authentic youth voices, and counter pro-vaping content on social media platforms where youth spend significant time. These coordinated efforts could help bridge the gap between interpersonal and societal disapproval while reinforcing protective social norms against both smoking and vaping.

This study has several methodological considerations. While the use of survey weights and adjustment for time-in-sample effects strengthen our findings, self-reported data may still be subject to recall and social desirability biases. While the cross-sectional design limits our ability to infer causality, it was chosen deliberately to examine changes in attitudes across age groups over time, rather than tracking individual changes as participants aged. This approach allows us to capture broader societal shifts in norms among youth of specific age ranges across different timepoints. Our analysis of the COVID-19 period (2020-2021) captures important shifts in social norms but may reflect temporary changes in social contexts rather than lasting trends.

Future research should build on existing qualitative research that has explored youth perspectives on peer influences and social identity associated with e-cigarette use. This could examine how different types of norms interact and evolve across diverse youth populations. Longitudinal mixed-methods studies combining survey data with in-depth interviews could explore the mechanisms behind the divergent trends we observed between descriptive and injunctive norms. Additionally, research examining how social media content and online communities shape different types of norms could inform targeted intervention strategies.

## Conclusion

This study demonstrates distinct trends in social norms regarding cigarette smoking and e-cigarette use among US youth during 2015-2021. While descriptive and injunctive norms consistently indicated the denormalization of cigarette smoking, norms regarding e-cigarette use showed more variation, particularly during the COVID-19 pandemic. The persistent gap between interpersonal and societal disapproval, especially for e-cigarettes, highlights the need for strengthening societal-level interventions while leveraging the strong protective influence of disapproval from family and close social circles. Coordinated efforts to align messaging across interpersonal and societal contexts could enhance the effectiveness of youth tobacco prevention strategies.

## Supplementary Material

ntaf120_suppl_Supplementary_Table_S1

## Data Availability

The data underlying this article are available in the Population Assessment of Tobacco and Health (PATH) Study Series database at www.icpsr.umich.edu/web/NAHDAP/series/606.
