# Supplementary material for: Trends in Social Norms Toward Cigarette Smoking and E-cigarette Use Among U.S. Youth Between 2015 and 2021
Source: Nicotine Tob Res. 2025 Jun 30;27(12):2168–76. doi: 10.1093/ntr/ntaf120 (PMC12641183; doi:10.1093/ntr/ntaf120)
Supplement: ntaf120_suppl_Supplementary_Table_S1 [file ntaf120_suppl_supplementary_table_s1.docx]

**Supplementary**

**Table S1. Associations between social norms and time (unadjusted).**

|  | Descriptive interpersonal norms | | | | | | |  | | Injunctive interpersonal norms | | | | | | |  | Injunctive societal norms | | | | | | | | |  |
| --- | --- | --- | --- | --- | --- | --- | --- | --- | --- | --- | --- | --- | --- | --- | --- | --- | --- | --- | --- | --- | --- | --- | --- | --- | --- | --- | --- |
|  | Having at least a few friends smoking | |  | | Having at least a few friends using e-cigarettes | | |  | | Thinking that people important to you view cigarette smoking negatively | | |  | Thinking that people important to you view e-cigarette use negatively | | |  | Thinking that most people disapprove of cigarette smoking | | | |  | Thinking that most people disapprove of e-cigarette use | | | |  |
| Time | OR (95% CI) | p-value | |  | | OR (95% CI) | p-value | |  | | OR (95% CI) | p-value | |  | OR (95% CI) | p-value | | |  | OR (95% CI) | p-value | | |  | OR (95% CI) | p-value | |
| Wave 3  (2015-2016) | 1.00 |  | |  | | 1.00 |  | |  | | 1.00 |  | |  | 1.00 |  | | |  | 1.00 |  | | |  | 1.00 |  | |
| Wave 4  (2016-2017) | 1.04 (0.96, 1.12) | 0.44 | |  | | **1.15 (1.07, 1.24)** | **0.01** | |  | | 1.03 (0.95, 1.11) | 0.53 | |  | 1.05 (0.98, 1.13) | 0.23 | | |  | 1.03 (0.96, 1.1) | 0.46 | | |  | 1.02 (0.96, 1.08) | 0.69 | |
| Wave 4.5  (2017-2018) | 1.08 (1, 1.18) | 0.09 | |  | | **2.72 (2.51, 2.95)** | **<.001** | |  | | **1.21 (1.12, 1.32)** | **<.001** | |  | 0.96 (0.89, 1.03) | 0.24 | | |  | **1.11 (1.03, 1.19)** | **0.01** | | |  | 0.95 (0.89, 1.01) | 0.08 | |
| Wave 5  (2018-2019) | 1.01 (0.93, 1.11) | 0.87 | |  | | **5.94 (5.43, 6.49)** | **<.001** | |  | | **1.34 (1.23, 1.46)** | **<.001** | |  | 0.98 (0.91, 1.06) | 0.49 | | |  | **1.13 (1.05, 1.22)** | **0.01** | | |  | 1 (0.94, 1.06) | 0.78 | |
| Wave 5.5  (2019-2020) | **0.8 (0.72, 0.9)** | **<.001** | |  | | **4.87 (4.4, 5.39)** | **<.001** | |  | | **2.95 (2.64, 3.29)** | **<.001** | |  | **2.32 (2.1, 2.57)** | **<.001** | | |  | **2.37 (2.16, 2.61)** | **<.001** | | |  | **2.34 (2.16, 2.53)** | **<.001** | |
| Wave 6  (2021) | **0.81 (0.72, 0.91)** | **<.001** | |  | | **7.45 (6.66, 8.33)** | **<.001** | |  | | **2.28 (2.03, 2.56)** | **<.001** | |  | **1.56 (1.41, 1.73)** | **<.001** | | |  | **1.78 (1.61, 1.97)** | **<.001** | | |  | **1.73 (1.59, 1.88)** | **<.001** | |

OR: Odds ratio, 95% CI: 95% confidence interval.

Data are from binary logistic regression analyses and weighted.

Bold values indicate statistical significance at p < .05.
